# Identity work of children with a parent with early-onset dementia in the Netherlands: Giving meaning through narrative construction

**DOI:** 10.1177/14713012211033494

**Published:** 2021-08-11

**Authors:** Silke Hoppe

**Affiliations:** Amsterdam Institute of Social Science Research, 1234 University of Amsterdam, Amsterdam, The Netherlands

**Keywords:** early-onset dementia, children, parent, identity work, narratives, the Netherlands

## Abstract

**Background:**

In the past years an increasing amount of research has been done on the experiences of adult children of a parent with early-onset dementia. However, little is still known about how the socio-cultural context influences the narratives of these children.

**Aim:**

This study aimed to provide insights into the far-reaching consequences of parental early-onset dementia for adult children in the Netherlands. It illustrates how the experiences of these adult children are shaped by the context they live in.

**Method:**

16 in-depth interviews were conducted with adult children of a parent with early-onset dementia in the Netherlands. The interviews offered the children space to reflect on the impact the illness of their parent had on them and their lives. The data were analysed using thematic analysis.

**Findings:**

This article illustrates that the comparative processes of relating to others' experiences help the children to reflect on the impact that their parent's illness has on their own lives, which in turn aids them in contextualising and making meaning out of their changing lives and relationships. This contextualization and recovery of meaning is shaped by three processes. The first concerns the ways these adult children draw comparisons between their own lives and experiences and those of their peers of the same age group. The second process entails comparative understandings of having a parent with early-onset dementia versus having a parent with late-onset dementia. The third process explores how having a parent with early-onset dementia compares to having a parent with other diseases. The processes of contextualisation which the adult children engage in are shaped by what the children perceive to be normal and thus also by their socio-cultural contexts.

**Conclusions:**

This article reveals how meaning is created in a constant interplay between the primary experiences of having an ill parent and the socio-cultural context in which the experiences take place. It illustrates how this context provides for particular narratives, which in turn shape how the children are able to give meaning to their experiences.

For many years, the perspectives of children with a parent who has early-onset dementia were neglected in academic literature (Gelman & Greer, 2011; Hall & Sikes, 2017; Hutchinson et al., 2016; Sikes & Hall, 2018). This gap has recently begun to be filled and research on these children is emerging as a vibrant field. Major research themes have included the experiences of these adult children, the impact that their parent’s illness has had on their lives and their resulting needs (Allen et al., 2009; Aslett et al., 2017; Barca et al., 2014; Gelman & Greer, 2011; Gelman & Rhames, 2018; Hall & Sikes, 2017, 2018, 2020; Johannessen et al., 2015; Millenaar et al., 2014; Sikes & Hall, 2018; Svanberg et al., 2010, 2011). Other authors have studied emotional well-being (Hutchinson et al. 2016), coping strategies and resilience (Allen et al., 2009; Johannessen et al., 2016; Svanberg et al. 2010) and healthcare service gaps (Barca et al., 2014; Gelman & Greer, 2011). Few authors, however, have focused on the socio-cultural context in which the adult children’s narratives about their parent’s illness and their experiences take place. One exception to this has been Hall and Sikes (2017) who discussed how broader cultural narratives of dementia may shape the extent to which children can conceptualize and express their experiences – especially when it comes to taboo topics such as admitting that one does not like the ill parent, that one would prefer if the parent were dead, or other experiences which may run counter to the ‘still the same person’ narrative surrounding people with dementia.

This article builds on the above described research to provide insights into the far-reaching consequences of parental early-onset dementia for adult children in the Netherlands. Further, it addresses a gap in the literature by demonstrating how the socio-cultural context shapes the narratives of such children. It shows how the children contextualize their experiences by comparing their situations to those of others. Looking for a story can help to make sense of an experience with illness (Frank, 1995; Kleinman, 1988). In times of adversity, re-examining one’s narrative can also be of help to maintaining a sense of identity (Bury, 2001: 264). Given that early-onset dementia is not a very well-known disease, adult children engage in identity work by relating their experiences to those of others. These comparative processes emerged from the interviews with adult children on which this article is based. Without being explicitly asked, children related their situation to that of others. This article illustrates that the comparative process of relating to others’ experiences helps the children to reflect on the impact their parent’s illness has on their own lives, which in turn aids them in contextualizing and making meaning out of their changing lives and relationships.

A story is always an edited version of reality and the storyteller chooses what to tell and what not to tell (Riessman, 1990: 1197). Likewise, the context from which one can draw comparisons to construct a story is boundless. The examples adult children use thus reveal which aspects of their experiences they want to emphasize and convey to outsiders.^1^ The aim of this article is not to compare the experiences of having a parent with early-onset dementia with other experiences. Instead, by following the children’s perspectives and by attempting to look at the situation through their eyes, we learn about what matters to them. The examples the children use often do not describe ‘real’ situations but rather contain idealized perceptions or simplifications which help them to think about their own situations.

Based on the empirical data that were collected, this article suggests that the contextualization and recovery of meaning that adult children with a parent who has early-onset dementia go through is shaped by three processes. The first concerns the ways these children draw comparisons between their own lives and experiences and those of their peers of the same age group. The second process entails comparative understandings of having a parent with early-onset dementia versus having a parent with late-onset dementia. The third process explores how having a parent with early-onset dementia compares to having a parent with other diseases. While illness and suffering are universal human experiences, they are coloured by cultural meaning. Cultural understandings can be ‘seen as tools (which both enable and constrain interpretive possibilities) available to navigate the ambiguity surrounding illness and other troubling experiences’ (Garro, 2000: 306). Narratives are likewise told within a cultural setting which offers specific forms of language, clichés and symbolic repertoire that serve to enable or restrict what can be expressed (Bury 2001: 278). Or in other words, ‘we tell our stories using the narrative forms available to us within our cultures’ (Sikes & Goodson 2017: 64).

This article demonstrates how the processes of contextualization which the adult children engage in are shaped by what the children perceive to be normal and thus also by the socio-cultural contexts in which they live. It reveals how meaning is created in a constant interplay between the primary experiences of having an ill parent and the socio-cultural context in which the experiences take place (c.f. Van Wijngaarden, 2019). Furthermore, it illustrates how this context provides for particular narratives, which in turn shape how the children are able to give meaning to their experiences.

## Early-onset dementia

People are diagnosed with early-onset dementia if they are under the age of 65. At first sight, the distinction between early-onset and late-onset dementia seems clear-cut. However, this distinction is arbitrary and not related to physical symptoms but instead is based on the age when people received a pension (Garre-Olmo et al., 2010). Although this distinction does not have any biological significance, it makes cultural sense because it demarcates a drastic change in people’s lives and expected societal roles. The Dutch Alzheimer’s Society estimates that of the 260,000 people with dementia in the Netherlands approximately 12,000 are under the age of 65. A Norwegian study estimated that about twenty-five percent of people with early-onset dementia in Norway have children (Haugen, 2012, in Hall & Sikes, 2020), though no specific statistics could be found regarding the respective Dutch situation.

Early-onset dementia comes in a variety of forms which include symptoms that differ from those which may manifest in dementia at a later age. The symptoms associated with early-onset dementia include social withdrawal, loss of empathy, disinhibition, exhibitionism, hyper-sexuality, aggression, impulsivity, short temper, obsessive compulsion, difficulty with planning and judgement making, visual disturbances, loss of physical functions, aphasia and communication disorders (Gelman & Rhames, 2018; Hall & Sikes, 2017; Sikes & Hall, 2018). Forgetfulness, which is often associated with late-onset dementia, is not a predominant symptom. As one can imagine, the symptoms of early-onset dementia thus have a different effect on the family than the symptoms of late-onset dementia. For instance, an aggressive parent can be more threatening to children than a forgetful parent.

Because the initial symptoms of early-onset dementia can resemble symptoms of depression or burn-out, it often takes up to twice as long to properly diagnose (Sikes & Hall, 2018). Van Vliet et al. (2013) found that the average time between the first symptoms and diagnosis for early-onset dementia is 4.4 years, compared to 2.8 years for late-onset dementia. This period often involves a lot of confusion, uncertainty and a disruption of ‘normal’ family practices (Hoppe, 2018; Sikes & Hall, 2018). It is a stressful period for even a grown person, and for a developing child, it can be even more intense. In order to mature emotionally and cognitively and to be able to lead an independent life, children need support from parents who can function reasonably well in their parental roles. Having a parent with early-onset dementia means both being deprived of this support and facing a changing relationship with that parent, who is to a decreasing degree able to fulfil their parental role (Johannessen et al., 2016).

In addition to the differences in prevalence and symptoms, the disease is ‘out of sync’ with a ‘normal’ life course (Aslett et al., 2017) and is perceived as untimely (Hall & Sikes, 2020). Parents are often working and raising a family and may have financial commitments such as mortgages to attend to (Aslett et al., 2017: 2). The person with dementia often loses the ability to provide an income for their family, which can lead to financial difficulties (Allen et al., 2009; Gelman & Rhames, 2020). Moreover, people with dementia struggle to increasing degrees to fulfil the roles of parent or spouse (Gelman & Rhames, 2018). This can manifest in no longer being able to help children with their homework, pick them up from soccer training, prepare meals or provide emotional support and care. Meanwhile, in a partnership the healthy parent might have to work less in order to care for the sick partner (Hutchinson et al., 2016) and also take over the tasks normally performed by the parent with dementia (Gelman & Rhames, 2020). Moreover, the healthy partner must balance these care needs with the needs of the children (Aslett et al., 2017) In summary, while some aspects (such as witnessing a family member change and deteriorate) are present in both early- and late-onset dementia, other aspects are tied to the specific moment in time in which the disease occurs and to the specific symptoms exhibited.

## Methods

The material for this article is based on 16 in-depth interviews with adult children of a parent with early-onset dementia in the Netherlands.^2^ The data were collected as part of my PhD research consisting of 41 qualitative interviews with seven people with early-onset dementia and thirty-six family members.^3^ Participants were recruited in a call on the National Alzheimer’s Society’s website and in three care institutions specialized in early-onset dementia, which circulated a folder in which I invited people to participate in my research. 6 of the 16 adult children actively reached out in response to my call. The 10 remaining children either joined the interview or agreed to be interviewed after I had spoken to their parent. The youngest child I interviewed was 19 and the oldest was 43.^4^ Most children lived in urban areas. I refer to children not as an age category, but rather to indicate a familial relation. The children mentioned in this article are all adults, but the majority of them still lived at home when their parent was diagnosed with early-onset dementia (see Table 1).

**Table 1.** Overview of adult children participating in research project.

Nr.	Name	Gender	Age upon first interview	Residence when first symptoms started	Own children	Age when parent displayed first symptoms	Age upon diagnosis	Age of parent when first symptoms appeared	Age of parent upon diagnosis	Time between diagnosis and interview (in years)	Residence of parent upon first interview
1	Lianne	Female	26	On her own	No	<20	20–25	50–55	55–60	2	Home
2	Thamara	Female	33	On her own	No	20–25	20–25	55–60	55–60	8	Nursing home
3	Klasina	Female	42	On her own	Yes	35–40	35–40	60–65	60–65	3	Nursing home
4	Koen	Male	21	With parent	No	<20	<20	50–55	50–55	6	Home
5	Andrea	Female	42	On her own	No	25–30	30–35	60–65	60–65	9	Nursing home
6	Juliette	Female	19	With parent	No	<20	<20	45–50	45–50	8	Nursing home
7	Charlotte	Female	36	With parent	Yes	<20	<20	45–50	50–55	16	Died
8	Aaike	Female	34	On her own	Yes	25–30	30–35	60–65	>65	2	Home
9	Maria	Female	36	On her own	Yes	25–30	25–30	55–60	55–60	8	Nursing home
10	Lejla	Female	26	With parent	No	<20	20–25	50–55	50–55	4	Nursing home
11	Maja	Female	21	With parent	No	<20	<20	50–55	50–55	4	Nursing home
12	Thijs	Male	28	On his own	Yes	<20	20–25	50–55	50–55	6	Home
13	Robin	Female	25	With parent	No	<20	<20	50–55	50–55	6	Home
14	Bodil	Female	43	On her own	Yes	35–40	40–45	55–60	60–65	1	Home
15	Gerben	Male	39	On his own	Yes	30–35	35–40	55–60	60–65	1	Home
16	Maaike	Female	≈36	On her own	No	25–30	25–30	55–60	60–65	7	Died

Three of the 16 interviews were done with pairs of siblings, seven were conducted with a parent, and six interviews with a single child alone. Additionally, three of the children were interviewed twice, either because I wanted to speak to them alone as well or because their circumstances changed (e.g. the parent was institutionalized). The majority of the interviews took place in the children’s homes, and the remainder took place in cafés. Interviews lasted between 1 and 3 h, 1.5 h on average.

In the interviews, I worked with a list of questions structured by topics such as the period before the diagnosis, the diagnosis and changes containing questions such as the first signs they noted, their thoughts and feelings around the changed behaviour of their parent, how they and their family reacted to the diagnosis, how their lives and their relationships have changed because of the illness of their parent, what helped them in the situation, how they looked into the future, how they thought society views dementia, and, finally, why they chose to participate in this research. I emphasized that I found it important that people shared what they consider important. In most cases, I just let people talk and the questions I had written down were addressed without me having to ask them (c.f. Goodson, 2017: 5). In other instances, people gave rather short answers and then I asked the questions I had written down. In the interviews, I did not ask the children to compare having a parent with early-onset dementia to having a parent with late-onset dementia or another disease. However, all children referred to situations of people they knew or to idealized situations in order to clarify their own situations. All interviews were recorded and then transcribed.

During open coding, I focussed on passages where children described the impact that their parent’s early-onset dementia had had on them. The themes of my topic list helped me to categorize the descriptions of the impact, distinguishing how the illness changed the children’s relationship to their parents and relationships with others as well as how it impacted their emotional well-being and their career choices. Many of these themes have been discussed in other literature. In my article, I add the role of context and argue that this context greatly shapes how these themes are experienced and narrated. Based on both primary and emerging themes, I then asked myself ‘why was the story told that way?’ (Bury, 2001: 281) and used the narratives to analyse the ways in which the children’s identity work is embedded into their socio-cultural context.

Ethical approval for this research was granted by the Amsterdam Institute for Social Science Research Ethics Committee before the start of fieldwork. This is a research ethics committee developed especially for social science research. The aim of the committee is not to check a checklist of points, but to challenge the researcher to think about ethical conduct and to make explicit how the project will lead to good research, not only in a methodological, but also in a social and ethical way. Prior to the interviews, participants were informed about the aims and objectives of the research. Verbal consent was obtained and recorded after the interview. Pseudonyms are used for all research participants in order to ensure anonymity.

Finding children who wanted to participate in my research was challenging. I asked several partners of a person with early-onset dementia whether I could also talk to their children and in many cases the children did not want to talk to me. Reasons were that the children were either busy with school or studies and/or that the topic was too painful for them to address. The reasons children did participate in this research were: wanting to contribute in some way, dealing with the feeling of powerlessness the illness brought up, contributing to more knowledge about early-onset dementia. I was aware that talking about their parent might bring up painful emotions and memories. Before the interview I therefore told my research participants that they did not have to discuss certain topics or answer specific questions if they did not want too. After the interview I asked how they experienced the interview. Experiences were positive. The children appreciated how I asked the questions and that the interview gave them the possibility to reflect on their experiences with the illness of their parent. Some were afraid beforehand that the interview would bring up painful emotions, but felt relieved after the interview. The timing of the interview was, however, important. Children found it easier to talk to me after they had some time to reflect on the situation themselves and get used to the new circumstances. This explains why the majority of the sick parents of the children who participated in this research were no longer living at home together with the child. I tried to make sure that my research participants left the interview in a good way. No participants left the interview upset. If that were the case, I would have contacted them a day later to check how they were doing. On average, I talked to the children almost 6 years after their parent was diagnosed. This gave them time to reflect on the situation, which shaped their narratives. I have included a table to illustrate how old the children were at the time of the diagnosis of their parent, whether they still lived at home and whether they had children of their own and when the interview took place.

I want to acknowledge that my presence shaped the interview and the narratives that were constructed. I take the position that narratives are not discovered, but are co-created during the interview (Fabian, 1990; Pool, 1994; Sikes & Goodson, 2017). The adult children I interviewed told ‘their story in a particular way for a particular purpose, guided by their understanding of the particular situation they are talking about, the self/identity/impression/image they want to present, and their assessment of how hearers will respond’ (Goodson, 2017: 8). McAdams (2017: 37) argues that we tell stories to bring ‘diverse elements into an integrated whole, organizing the multiple and conflicting facets of our lives within a narrative framework, which connects past, present, and an anticipated future and confers upon our lives a sense of inner sameness and social continuity – indeed an identity’. The lived reality is more messy, complex and ambiguous than the story that describes it (Baldwin, 2017: 537).

Writing down the stories poses the danger of freezing and fixing them, not allowing them to change in the future (Sikes, 2017: 411). Therefore, I want to emphasize that the material discussed in this article is not static. The comparative processes I describe took place in every interview, but how the children made comparisons is time- and context-sensitive. Or as Sikes (2017: 411) convincingly states: ‘Lives and making sense of them are both works in progress’. The adult children’s narratives are tied to their life experiences and those change constantly.

## Findings

As introduced above, the findings of my interviews illustrated that adult children of a parent with early-onset dementia struggle with questions of identity and engage in comparative processes to give meaning to their situations. The type of identity work which children engage in emerged organically from the interview data. In the following sections, I present empirical examples which illustrate the three-part comparative processes I described above. One important question I considered was whether these comparative processes were limited to the interview or also occurred outside of it. Identity work through comparison is an iterative process, meaning that the children share their experiences with others and, based on the reactions they receive, adapt both their narratives and their own interpretations of the situation. This process became visible in the empirical data. Children mentioned how they told their stories to others and how this made them realize how bad their situations really were (i.e. how young their parents were or how little others knew about early-onset dementia). Reactions from others likewise made them re-evaluate and reinterpret their situations and sometimes attribute new meanings to their experiences. I take the interview to be a snapshot of this process. The interview was a space in which children could attribute meaning to the situation because they were given the space to reflect on their experiences, but this process began before the interview took place and continued afterwards.

### Having a parent with early-onset dementia versus imagining growing up normally

All the children I interviewed expressed that their relationship to the parent with early-onset dementia changed after the onset of the disease.

Charlotte (36): Well, your father should care for you and that is no longer the case. So we care for our Dad and he has never been the type of father who helps with jobs around the house. You care for your father instead of your father caring for you and that’s not how it should be. In your head it is very weird, because that’s not how it should be.

### Most reported that roles became reversed

Lejla (26): When he still lived at home, I took over quite a lot of care tasks, I felt like I was being a kind of parent to him. I thought, that’s not what a twenty-year-old should have to deal with. I have my job on the side, I have school. I want to occupy myself with other things, maybe that sounds shitty…

Maja (21): No, I get it. Because that was also the case with our mother, we had to be there for her. We basically had no one.

Lejla (26): Yes, you are coaching or caring for your parents. Reversed roles. That was difficult.

Maja: Yes, that is very heavy.

When discussing what is ‘normal’, adult children often referred to a culturally specific ideal, which is constituted in a complex web of norms and values. The children I interviewed compared themselves to peers of a similar age who received support from both parents and thus were able to more fully enjoy their lives. They thereby emphasized the contrast between themselves and their age peers.

Juliette, who was 19 at the time of the interview, had twice decided to temporarily live with a friend’s family for several months because she could no longer cope with the situation at home.

Juliette (19): Actually, I don’t have a mother, never had, because before she cared a lot for my [disabled] brother, so I received very little attention. So actually, I never had a mother. Physically yes, but not someone who had cookies ready when I returned from school, who asked about school, ‘how are you?’, ‘how was school?’, she never did that, no. This really is because of the Alzheimer’s, because you cannot put yourself in the shoes of another.

Juliette’s statement that she never really had a mother is based on her understandings of what a mother should do and should not do. She thus has a cultural model in her mind which she uses as a reference. One could say that in the interview Juliette refers to an idealized view of a mother in the Netherlands. This ideal is comprised of loving parents who care for their children, are available to their children, and always have the best interests of their children in mind.

Another aspect that the children mentioned was how their plans for the future had been shaped by their parents’ illness. In the case of Juliette, the stress at home meant that it took her 2 years longer to finish school than her peers and that she chose to study close to her hometown because she did not want her father to live all by himself. Robin and Koen also adapted their plans to the situation at home.

Robin (25): I notice that within our family, that you live your life around the illness. Dad’s illness determined a lot of activities and everyone’s life, because Koen is studying in another city and he probably would have liked to live there as well. And I wanted to go to the capital after finishing my studies and go on a trip. For about ten years you adapt your choices, and that is your own decision, but I notice that it has determined all of our lives.

Koen (21): For sure, also with moving out. I found it very difficult to leave the house. It felt like leaving a sinking ship. But it just was not healthy anymore to stay longer…

We can see that Koen is torn between his wish to lead his own life, on the one hand, and his loyalty towards his family on the other (for a more detailed analysis, see Hoppe, 2020). During the interview he stated that he and his mother were going against mainstream ideas because they wanted to care for their family member with dementia for as long as possible.

Freedom, individuality and independence are important values in the Netherlands and, furthermore, there is an increasing emphasis on individual choice (Giddens, 1991). These factors shape the perception of how the children’s parent’s illness shapes their own lives. The children are clearly influenced by their parents’ illness, but whether they perceive it as a restriction, abnormal or unhealthy depends to a large degree on the socio-cultural context in which they grow up.

### Having a parent with early-onset dementia versus imagining having a parent with late-onset dementia

Most children commented on the life stage in which their parent’s illness took place and compared it to dementia occurring in later life. Upon closer analysis, many different dimensions are revealed in their comparisons. The underlying motivation for the comparison seems to be to assess the severity of the situation.

Lejla (26): We were really young when it happened, also if you tell it to people, then I think ‘wow, but you lost your father at a really young age’.

Maja (21): Sometimes it happens to me, that only then do I realise how intense it all is.

Lejla (26): Yes, sometimes I think I will never get to know my father as a grown-up person. I just knew my father as a child, a teenager, and adolescent.

Although their father was still alive, to the sisters it felt as if he were already dead. In this statement, the severity of the situation is predominately linked to the phase in which the illness developed and not so much to the symptoms themselves. Lejla’s last statement also reveals the idea that one knows a person differently based on the life stage one occupies. One could say that because of the dementia, she mainly encountered her father in his role as father and she did not have to chance to meet him independently from that role.

In all interviews, research participants stated that the illness did not fit the life stage in which it occurred.

Bodil (43): I struggle with the fact that my mother is young and has it. I can imagine that if she were 80 and she would become forgetful, that would fit better with her age. I don’t know whether I would accept that better, because you still have to say goodbye to a person you love…

The severity of the illness is linked to the age of the person concerned. But how dementia is experienced is not only related to the life stage, but also to the relationship one has with the person and the role that person fulfils in one’s life.

Some children also pointed out that there can be large differences with regard to age within early-onset dementia.

Juliette (19): Lately I read something about Alzheimer’s in younger people and then they referred to someone who was 65. And I thought ‘65. I wish my mother got it at 65, then I would have moved out a long time ago. Maybe already had my own family’. So, it is a very different story, hearing that you have Alzheimer’s when you are 50. That is real early-onset dementia. I can get pretty angry about this. Some people do not take into consideration that there are people who are younger than 65.

The severity of the illness in this case is not only related to the life stage of the sick person, but also to one’s own life phase. Juliette basically says that if she had her own family, her mother’s illness would have impacted her differently as she would have been less dependent on her mother and on her mother’s care and support. Moreover, her statement reveals that she does not feel understood by her environment and that this lack of recognition makes her angry (see Hoppe, 2018).

Next to the life phase in which the illness occurs, symptoms were also mentioned as a distinguishing feature.

Maja (21): For a long time, he was the youngest. With the others I notice, they are just elderly people who have Alzheimer’s or something else. And you can just talk to them. We talk about the weather, but that’s still possible.

Lejla (26): With him, it was no longer possible relatively early in the trajectory.

Maja (21) Yes, you couldn’t have a conversation with him.

Lejla (26): That is true, he really is different from the other residents. The elder people who also have Alzheimer’s still look decent.

It is thus not only the life phase in which the illness occurs that shapes the experience, but also the type and severity of symptoms. In younger people, it is not so much forgetfulness but rather neuropsychiatric symptoms which stand at the foreground (van Vliet et al., 2010). Again, what counts is likely not so much what the symptoms are but how the symptoms shape the child’s relationship to their sick parent. Likewise, how the parent’s symptoms are experienced by the child depends on the kind of relationship they had and what is valued. For the sisters, looking neat seems to be important and having a father who is no longer ‘clean’ affects them differently than the idea of a father who, for example, can no longer walk.

The empirical material in this section demonstrates that the perception of the illness is shaped by many different aspects which are not necessarily connected to the illness itself but much more to the life phase in which the illness occurs.

### Having a parent with early-onset dementia versus imagining having a parent with another disease

In interviews, early-onset dementia was often compared to other diseases. Strikingly, the majority of the children interviewed referred to cancer in these comparisons. They used their imagined experience of having a parent with cancer to demarcate what it is like to have a parent with early-onset dementia, evoking certain characteristics of cancer and leaving out others.

Juliette (19): This is something I have been struggling with in the past months, and also discussed with a psychologist. Because I cannot really say goodbye. I can’t say ‘Hey Mom, I feel very bad that you are so sick, I find it horrible, I really miss you’. Other people who have sick parents, for example, who have cancer, their mind is still there, they are only physically sick. And this is the other way around. This is terrible, because that’s not what you want. You don’t want to say goodbye, but if you have to say goodbye, you want to do it solemnly, properly, and lovingly. But well, she cannot hear me, she doesn’t understand me, so how do you want to do it?

The reference to cancer was used to emphasize that it is important whether one can still say goodbye to one’s parent or not. The quote further illustrates how a delineation between body and mind is drawn. For Juliette, saying goodbye is a dialogue and requires a shared reality or consciousness – in other words, the presence of both body and mind.

Another aspect which was compared to cancer in interviews was the presence or absence of pain.

Charlotte (36): Until he got really sick, he was never really in pain, pain in his head, but at a certain point you are no longer aware of it. You also have people with cancer, who lie in bed for a year and contort with pain. Look, in the end, he had no pain, well, a different kind of pain, he became less motoric and walked a bit crooked. I hope that he was happy in his own way. Well, you can wonder about that, but you won’t get an answer anymore.

This fragment highlights that children can experience their parent’s illness very differently. Whereas Juliette points out an aspect that, according to her, is more difficult when it comes to having a parent with dementia, Charlotte relativizes her experience and instead points to cases that are worse than that of her father. Of course, suffering or perception of the severity of an illness cannot be compared. Likewise, people with dementia might be suffering from pain without being able to communicate that pain. Clinical studies indicate that undertreatment of pain is common in dementia patients (Scherder et al., 2009) and dreaded by family members (Lemos Dekker, 2018), but none of the children brought this up in interviews. Charlotte, however, mentioned another kind of pain: not knowing whether her father was happy. From her perspective, in cases of cancer this can be asked and one can receive an answer. In the case of dementia, receiving an answer might be impossible.

The narratives of these children should not be read as direct representations of reality. Instead, they provide insight into how the children give meaning to their situations and the process of doing so by comparing and differentiating their own situations to the situations they imagine of others. References to cancer were not only made to discuss the presence or absence of pain, but also the visibility of the disease.

Robin (25): It is difficult, because for example cancer—everyone knows someone with cancer in his environment. It’s a very different process. But also a much more familiar process to people of our age, I think.

Koen (21): And also it is much more discussed in the media. If you see how people at school or whatever, jump onto it, if one of the parents has cancer, that is very different from dementia. Probably also because dementia is such a long-running process…

Robin (25): But also a less familiar one I think…

In the children’s eyes, cancer is not only better known, but also taken more seriously than dementia.

Thamara (33): 100,000 jokes are made, in the media, in comics, it’s everywhere—Alzheimer’s, dementia. Often, it is treated as a joke. It’s funny, isn’t it? But I mean, do that with cancer and then half of the world will protest if you make jokes about that. But with Alzheimer’s it is just accepted. That’s what I find frustrating.

The lack of media attention combined with negative representations of people with dementia results in those within the social environments of these children (consisting of relatives, friends, teachers, etc.) not necessarily having a realistic picture of the situation. It can therefore be harder for the children to access understanding and support.

In other cases, children drew on cancer to point out not contrasts but similarities. Thamara stated that if her mother had breast cancer she would probably check her own breast every day, just as she tries to stay healthy in order to avoid getting dementia in the future. Juliette pointed out that because her boyfriend had lost his father to cancer, he better understands her situation.

## Discussion

In this article, the narratives of children who have a parent with early-onset dementia in the Netherlands have been analysed. It has been argued that because early-onset dementia is a relatively unknown condition, children struggle to relate to broader cultural narratives in order to tell their stories. As a result, they instead construct their stories by relating to culturally salient experiences of others. Their constructions are shaped by three comparative processes: relating to children with healthy parents, relating to having a parent with late-onset dementia and relating to having a parent with another disease.

Various scholars have pointed out how illness experiences are shaped by not only individual circumstances but also by the broader socio-cultural, structural and historical contexts. To put it differently, ‘different illness experiences become available at different times’ (Klawiter, 2004: 845). Klawiter (2004) describes how a woman who was diagnosed with breast cancer in two different decades experienced her illness. When the woman was first diagnosed in 1979, the broader context was characterized by the sovereign power of physicians, the isolation and disempowerment of patients and the invisibility of women with breast cancer in the public domain (resulting in the absence of a group identity). In the 1990s, when she was diagnosed for a second time, breast cancer had become a visible presence in the public domain, breast cancer survivors were seen as heroes rather than as victims, and patients had access to education workshops and support groups and were treated by a healthcare team consisting of a range of professionals. ‘Instead of feeling isolated and powerless, as she had in 1979, Clara felt like the captain of a well-functioning team dedicated to aiding and assisting her treatment and recovery’ (Klawiter, 2004: 861). The broader socio-cultural context thus shaped both her experiences and the language and terminology that were available to her.

Similarly, support groups can shape illness experiences by constructing a collective illness identity and giving patients a vocabulary with which to talk about their illness. This subsequently serves as the basis for individual identity work. Koski (2014) has demonstrated in the case of eating disorder support groups that collective narratives have significant power; when eating disorders were constructed as a chronic condition rooted in the self and uncontrollable, the framing was shown to have unanticipated and potentially adverse consequences for participants who internalized it. Political discourse can also shape how groups of people are viewed and treated. Likewise, policy discourses are shaped by collective narratives and in turn play a role in shaping these narratives. In the case of dementia, analysing policy narratives can provide insight into how and whether people with dementia can practice citizenship and which services they can expect from welfare organizations (Nedlund & Nordh, 2015: 124).

Given the impact of these collective factors on identity work, mapping the context in which these children live and distinguishing aspects that can shape their experiences and narratives becomes important. As mentioned above, it is considered normal in the Netherlands for children to be cared for by their parents and raised to live independent lives. When children express that they have lost their parent while the parent is still alive, they are describing not the biological but the social death of their parent (Glaser and Strauss, 1966: 108, in Sweeting & Gilhooly, 1997: 94). In other words, the parent is no longer able to fulfil their culturally prescribed role. The extent to which this role is fulfilled is defined within this cultural context, but can vary considerably across individual understanding. This point is important to the present discussion because it underlines that the impact of early-onset dementia in a parent is experienced differently depending on the parent–child relationship. For example, if a child’s relationship to their parent is characterized predominately by physical contact and proximity, the impact of the disease will be different than if the relationship is based mainly on verbal exchange.

In the past decade, the Netherlands has seen a shift from a welfare state model – in which it was normal for older people to be moved into a home for the elderly – to a participation society, in which family members and neighbours are expected to care for the sick and elderly (Da Roit & de Klerk, 2014; Vollenberg et al., 2013). In spite of this shift, the ideal is still that children should have a carefree childhood and youth (Hall & Sikes, 2020) in which they should not carry too much responsibility and should not be confronted with too much burden. Thus, overall, the children I interviewed felt that it was not normal that they were burdened and restricted by their parent’s illness.

Cultural narratives surrounding dementia further complicate the children’s navigation of their parent’s illness. Across cultures, dementia is often seen either as a natural part of the ageing process or as the result of a brain disease (Hillman & Latimer, 2017: 1). Within the Western world, dementia is approached in two contrasting ways. On the one hand, cultural narratives on dementia often describe dementia as a terrifying disease and on the other hand people argue for a more humane approach to dementia.

Dementia personifies what people fear most about growing old (Lock, 2014, in Hillman & Latimer, 2017: 1). Peel (2014) analysed media representations of dementia and found that dementia was represented in either catastrophic terms or in terms of individualistic recommendations to keep the illness at bay, thereby implying personal responsibility for the condition. People with Alzheimer’s disease are often depicted as zombies in scholarly and popular literature (Behuniak, 2011), such that one poll found that people fear dementia even more than death (Brooks, 2011: 42, in Peel, 2014: 890). Researchers have coined the term ‘dementia worry’, finding that ‘there is emerging evidence that DW is a relatively widespread and probably increasing phenomenon in Western societies and that DW is at the top of all disease worries’ (Kessler et al., 2012: 277).

In contrast to the gloomy view described above, there are also movements that focus on living well with dementia and which support the view that people with dementia are still the same person they were before the onset of the disease (Hall & Sikes, 2017). As is evident from these examples, the cultural meanings attached to dementia are very diverse (see Nedlund & Nordh, 2015). ‘Meanings of dementia are interpreted, embodied, or resisted by people in their social context, and these processes are shaped according to their social location (gender, social class, and ethnicity) and their individual biography’ (Hillman & Latimer, 2017: 1).

Instead of referring to catastrophic depictions of dementia, the children I interviewed referred to a view in which a decline in health and cognitive abilities is seen as a normal part of ageing (Higgs & Gilleard, 2015: 2) and where the death of older people is considered natural or appropriate (Sweeting & Gilhooly, 1997: 96). They hence increased the contrast they were drawing between dementia in elderly people versus dementia in younger people. ‘When things happen out of order at unexpected times, they seem to have a greater impact than when events happen “on time”’ (Hagestad, 1990, in Barca et al. (2014): 1941). A central element of the narratives of these children is that the illness of their parent did not happen ‘on time’. However, the experience of the illness is not so much shaped by the age of the parent, but by the perception of age and by the relationship. Indeed, if a parent was to have children later in life and be diagnosed at the age of 64, their children might still perceive the illness as happening ‘too soon’. Tolhurst (2016: 13) states that differencing young people with dementia from old people has advantages, in that it ‘distinguishes them from societally undervalued and stigmatised old people’. Based on the interviews with children, I do not get the impression that this is the reason why they emphasized the distinction. Yet, it is something to be cautious about when writing about early-onset dementia as highlighting the specific needs of young people with dementia and their family members can be a form of ageism, implicitly accepting that older people have reduced social roles.

Hall and Sikes (2020) have argued that these children are in a liminal phase until their parent’s death because their parents are both present and absent. The authors show how the lives of these children are somehow put on hold and as such that the children fall between socially recognized categories. As their parent is still alive, they do not receive the support and social status they would receive if the parent was dead. Yet, they already mourn the parent they knew or in other words, they mourn the social death of their parent.

While the discourse that dementia patients are ‘still the same person’ is relevant for person-centred care, in which attempts are made to maintain the personhood of individuals with dementia (Smebye & Kirkevold, 2013) and in which the needs of the patient are paramount (Tolhurst et al., 2014), the children did not reference this narrative. Sikes and Hall (2016) have demonstrated how going against this narrative can work as a safety mechanism for the children, because they can believe that their parent would never act in the ways that they are if they were not sick. In the interviews, children protected the identities of their parents by emphasizing that certain behaviour was caused by the illness or by stating that their parent could not do anything about it. They delineated a difference between the parent before the onset of the illness and after. Tolhurst (2016) argue that framing dementia in positive terms generates a zero-sum situation, in which the dignity of the person with dementia is protected, but the difficulties of family members are being invalidated. As suggested above, I argue that how the children narrate their experiences depends on their needs and on their audience. In the interviews, the majority of children wanted to get across how much their parent’s illness had changed their lives and how difficult the situation was for them. Therefore, they comparatively referenced situations which they considered to be ‘less bad’ so that the contrast would become clear. No child I interviewed made a comparison to age peers growing up with a single parent. This is likely because while such as situation might have similarities to their own, there would be a risk that the listener might not understand the pain of losing the person they knew. In their daily lives, the children are confronted with people’s stereotypical views on dementia and resulting lack of understanding, which, as I have argued elsewhere (Hoppe, 2018), can add to their struggles and make their situations more difficult.

None of the children I interviewed participated in a support group, although some stated that they would have appreciated contact with age peers. The only groups that were available to them were targeted at family members of older people with dementia and the children felt that the issues being discussed were too greatly removed from the problems they encountered. They were not ‘exposed’ to a collective narrative on early-onset dementia. As a consequence, they individually constructed their own narratives, drawing from examples in their familiar environment. Despite these individual constructions, in this article I have shown how the narratives still all centred around patterns of comparisons with children with healthy parents, children whose parents have late-onset dementia and children whose parents have another disease. Their experiences and narrations of their parent’s illness also depended on other factors such as gender (of the child, but also of the parent), age, ethnicity and education. The ways in which these factors shaped the narratives and whether differences of importance could be ascribed to these factors is outside the scope of the present study, but I believe that they can be understood to have coloured the narratives nonetheless. More research is necessary to analyse the narratives from this point of view.

In this article, three comparative processes that children used to communicate what it means to have a parent with early-onset dementia and to give meaning to their experiences have been discussed. Analysing these processes of identity work and what children considered important to communicate pointed to the specificities of having a parent with early-onset dementia and how these specificities impacted the children. Furthermore, this article has shown how the children’s narratives were imbedded in and shaped by the socio-cultural context and how children made use of this context to give meaning to their situations. Goodson (2017: 5) argues that ‘Narratives […] are best when fully “located” in their time and place’. This article has provided insight into how narrative and context are related and intertwined. On a micro scale, one can say that their narratives also contributed to the context (cf. Hillman et al., 2018). Research on the experiences and narratives of children who have a parent with early-onset dementia in other parts of the world is needed to deepen our understandings of the extent and forms which this socio-cultural shaping of experiences and narratives can take.

## Notes

1 When asking my research participants about the changes they experienced, the majority made comparisons to age peers and did not make a comparison to how the situation was prior to the diagnosis of the parent. One possible explanation is that in many cases the onset of the illness happened several years ago and memories of how the relationship used to be before the onset of the illness were overshadowed by changes in the present.

2 In one interview, I discovered that the parent with dementia was above 65 when being diagnosed. I still included the participant in my study because she self-identified as a child of someone with early-onset dementia. Material of the interview with her is not included in this article.

3 This PhD project is part of the Partnership for Long-term Care and Dementia based at the University of Amsterdam. The partnership was set up by a collaboration of different parties and brought together the expertise of academic researchers, stakeholders and care providers with the aim to improve the quality of life for people with dementia and the work quality of caregivers in the Netherlands. Together with my colleagues all working on dementia I set up dialogue evenings in which, on a regular basis, we initially discussed our findings with people in the field and later learned together with them about dementia and its impact (for a detailed description of this process, see Hoppe et al., 2019). We also organized a collaborative conference in which we discussed dementia together with people with dementia, family members, care managers and care workers (see Amsterdam Care Collective, 2018). The collaborative projects I did with my colleagues were not specifically about early-onset dementia, but my contributions were. In addition, I gave presentations at the Dutch Alzheimer Society and a network organization bring together care organizations specialized in early-onset dementia in the Netherlands.

4 The children I talked to were all adults and thus older than participants of other studies (e.g. Gelman & Greer, 2011, Gelman & Rhames, 2020; Hall & Sikes, 2017, 2018). Younger children may have different expectations of parental support from older children. As a consequence, the age of the child upon the beginning of the parent’s symptoms may shape how the parent’s illness is experienced and what meaning children ascribe to it.
